# PALLIATIVE GASTRECTOMY VERSUS GASTRIC BYPASS FOR SYMPTOMATIC CLINICAL STAGE IV GASTRIC CANCER: A PROPENSITY SCORE MATCHING ANALYSIS

**DOI:** 10.1590/0102-672020230072e1790

**Published:** 2024-02-05

**Authors:** Sterphany Ohana Soares Azevêdo PINTO, Marina Alessandra PEREIRA, Ulysses RIBEIRO, Luiz Augusto Carneiro D’ALBUQUERQUE, Marcus Fernando Kodama Pertille RAMOS

**Affiliations:** 1 Universidade de Sao Paulo, Faculty of Medicina, Cancer Institute, Hospital de Clinicas, Department of Gastroenterology – São Paulo (SP), Brazil.

**Keywords:** Stomach Neoplasms, Gastrectomy, Gastric Bypass, Palliative Care, Survival Analysis, Neoplasias Gástricas, Gastrectomia, Derivação Gástrica, Cuidados Paliativos, Análise de Sobrevida

## Abstract

**BACKGROUND:**

Patients with clinical stage IV gastric cancer may require palliative procedures to manage complications such as obstruction. However, there is no consensus on whether performing palliative gastrectomy compared to gastric bypass brings benefits in terms of survival.

**AIMS:**

To compare the overall survival of patients with distal obstructive gastric cancer undergoing palliative surgical treatment, using propensity score matching analysis.

**METHODS:**

Patients who underwent palliative bypass surgery (gastrojejunostomy or partitioning) and resection between the years 2009 and 2023 were retrospectively selected. Initial and postoperative clinicopathological variables were collected.

**RESULTS:**

150 patients were initially included. The derived group (n=91) presented more locally invasive disease (p<0.01), greater degree of obstruction (p<0.01), and worse clinical status (p<0.01), while the resected ones (n= 59) presented more distant metastasis (p<0.01). After matching, 35 patients remained in each group. There was no difference in the incidence of postoperative complications, but the derived group had higher 90-day mortality (p<0.01). Overall survival was 16.9 and 4.5 months for the resected and derived groups, respectively (p<0.01). After multivariate analysis, hypoalbuminemia (hazard ratio — HR=2.02, 95% confidence interval — 95%CI 1.17–3.48; p=0.01), absence of adjuvant chemotherapy (HR=5.97; 95%CI 3.03–11.7; p<0.01), and gastric bypass (HR=3,28; 95%CI 1.8–5.95; p<0.01) were associated with worse survival.

**CONCLUSIONS:**

Palliative gastrectomy was associated with greater survival and lower postoperative morbidity compared to gastric bypass. This may be due to better local control of the disease, with lower risks of complications and better effectiveness of chemotherapy.

## RESUMO

**RACIONAL:**

Pacientes com câncer gástrico estádio clínico IV podem necessitar de procedimentos paliativos para o manejo de complicações como a obstrução. Contudo, não há consenso se a realização de gastrectomia paliativa em comparação à derivação gástrica traz benefícios em termos de sobrevida.

**OBJETIVOS:**

Comparar a sobrevida global de pacientes com câncer gástrico obstrutivo distal submetidos a tratamento cirúrgico paliativo, empregando a análise com pareamento por escore de propensão.

**MÉTODOS:**

Foram selecionados retrospectivamente pacientes submetidos à cirurgia paliativa de derivação (gastrojejunostomia ou partição) e ressecção entre os anos de 2009 e 2023. Variáveis clínico-patológicas iniciais e pós-operatórias foram coletadas.

**RESULTADOS:**

Foram inicialmente incluídos 150 pacientes. O grupo derivado (n=91) apresentou mais doença localmente invasiva (p<0,01), maior garu de obstrução (p<0,01) e pior *status* clínico (p<0,01), enquanto os gastrectomizados (n=59) mais metástase à distância (p<0,01). Após o pareamento, restaram 35 pacientes em cada grupo. Não houve diferença na incidência de complicações pós-operatórias, mas o grupo derivado apresentou maior mortalidade em 90 dias (p<0,01). A sobrevida global foi de 16,9 e 4,5 meses para o grupo ressecado e derivado, respectivamente (p<0,01). Após análise multivariada, hipoalbuminemia (HR=2,02; IC95% 1,17–3,48; p=0,01), ausência de quimioterapia adjuvante (HR=5,97; IC95% 3,03–11,7; p<0,01) e bypass gástrico (HR =3,28; IC95% 1,8–5,95; p<0,01) foram associados a pior sobrevida.

**CONCLUSÕES:**

A gastrectomia paliativa esteve associada a maior sobrevida e menor morbidade pós-operatória quando comparada à derivação gástrica. Isto pode se dever a um melhor controle local da doença, com menores riscos de complicações e melhor efetividade da quimioterapia.

## INTRODUCTION

Gastric cancer (GC) is the 5^th^ leading cause of cancer death worldwide and accounts for 9% of all cancer-related deaths in Brazil^20,21^. Unfortunately, in developing countries, up to 50% of patients are diagnosed with clinical stage IV at the time of first presentation^4,30,33^. Clinical stage IV includes locally unresectable T4b tumors or metastatic disease^2^. The standard palliative therapy for those cases is systemic chemotherapy with some cases being able to undergo conversion surgery even with curative intent^2^.

However, chemotherapy is usually not feasible when the patient is highly symptomatic and presents gastric outlet obstruction (GOO) or bleeding. In these situations, surgical procedures such as gastric resection or gastric bypass may be performed^9,10^.

Gastrectomy has the advantage of completely removing the tumor, preventing the occurrence of future local complications with a possible benefit in the action of systemic treatment by reducing the tumor burden^19^. However, there is a risk of complications related to the anastomoses and duodenal stump^5^. It is worth noting that most patients in this situation have significant weight loss with malnutrition and clinical frailty^6^. As an alternative, in cases where the lesions are unresectable or the patient is at high surgical risk, bypass procedures such as gastrojejunostomy and gastric partitioning can be used. Both are easier to perform, but by not removing the primary tumor, the possibility of future complications remains^26,29^.

The question of whether palliative gastrectomy improves overall survival has long been debated^3,7,10,12,16,17,25,31,32,33^. The doubt as to whether there is a real benefit from the reduction of local tumor volume or whether systemic disease control with chemotherapy is the main prognostic factor persists^15,16,18,23,24^.

Thus, these study aims were to analyze and compare the overall survival between symptomatic stage IV gastric cancer patients that underwent palliative gastrectomy and gastric bypass, using the propensity score matching method to control for selection bias.

## METHODS

### Study design and sampling

A retrospective cohort study was conducted after approval by the Hospital Ethics Committee (NP1681/20) and the National Ethics Board (Certificate of Presentation for Ethical Appreciation — CAAE: 31626220.8.0000.0068). We selected all consecutive patients who underwent surgical intervention with clinical stage IV GC between 2009 and 2023 at our institution.

The inclusion criteria were as follows:

1. Histological confirmation of gastric adenocarcinoma;
2. Clinical stage IV GC; and
3. Palliative procedure for GC-related symptoms.

Patients that have had a recurrent tumor, gastric perforation, performed only jejunostomy, or underwent conversion therapy were excluded. As palliative surgery, we included palliative gastrectomy (total or subtotal) and gastric bypass (gastric partitioning or gastrojejunostomy). All cases were operated in a high-volume center by specialized surgeons. The surgical technique was performed in accordance with the Japanese Gastric Cancer guidelines^11^.

### Clinical variables

The baseline clinical variables included were age, sex, clinical TNM (cTNM), number of metastatic sites, Charlson Comorbidity Index (CCI) without the inclusion of GC as a comorbidity, the American Society of Anesthesiologists classification system (ASA), preoperative Karnofsky Performance Status (KPS), preoperative Eastern Cooperative Oncologic Group performance score (ECOG), albumin (g/dL), hemoglobin (g/dL), body mass index (BMI), and gastric outlet obstruction scoring system (GOOSS). The GOOSS is defined as 0 for no oral intake, 1 for liquids only, 2 for soft solids, and 3 for low residue or full diet^1^.

The outcomes evaluated included postoperative complications (POC) according to the Clavien-Dindo classification, length of hospital stay, administration of post-operative palliative chemotherapy, 30 and 90-day mortality and overall survival (OS).

### Statistical analysis

Descriptive statistics were presented as frequencies, for categorical variables and mean with standard deviation (±SD), or median with interquartile ranges (IQR), for continuous variables. Data distribution was assessed by the Kolmogorov-Smirnov test. The ꭓ^2^ test and Fisher’s exact test were used to compare parametric categorical variables, whereas the Mann-Whitney U test and median test were applied to the non-parametric continuous variables.

To control for selection bias and ensure that the groups could be compared regarding the treatment used, propensity score matching was applied, with a 1:1 nearest neighbor matching and a caliper of 0.01. For matching, the covariates selected were age, sex, nodal clinical staging (cN), presence of metastasis (cM), CCI, ECOG, and preoperative albumin and hemoglobin.

The overall survival (OS) was calculated from the date of the surgery until the patient’s death. Survival was estimated using the Kaplan-Meier method, and the difference between survival curves was evaluated with the log-rank test. The Cox proportional hazards regression model was conducted to define which factors were related to the researched outcome. For the univariate analysis, we selected covariates that are known to influence the outcome, based on background clinical knowledge. Variables with a p-value < 0.2 were entered in the multivariate analysis. A p-value < 0.05 was considered statistically significant, and the effect size of the covariates between subsets was assessed using the HR. All statistical analyses were performed using the IBM Statistical Package for the Social Sciences — SPSS version 25.0 (IBM SPSS, Armonk, NY).

## RESULTS

**Figure 1:**
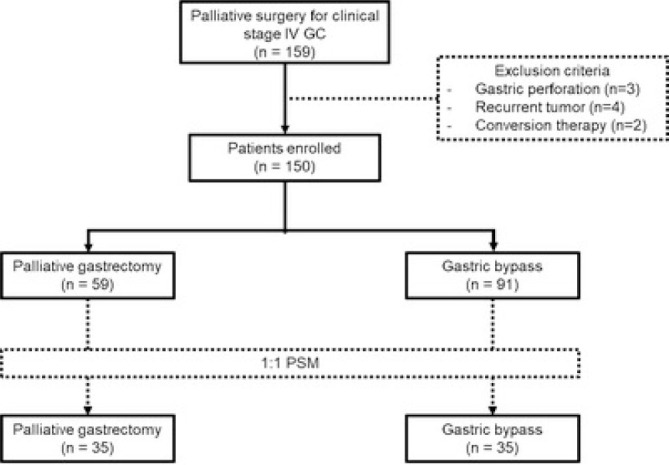
Flowchart of the study.

From 2009 to 2023, 159 patients with advanced GC underwent palliative surgery at our institution and met the inclusion criteria. Of these, three were excluded due to perforation, four had a recurrent tumor and two underwent conversion therapy. The remaining 150 patients were analysed in this study. The bypass procedure was performed on 91 patients and, among them, 39 had gastric partitioning (42.9%) and 52 had gastrojejunostomy (57.1%). Palliative gastrectomy was performed in 59 patients, with 35 subtotal (59.3%) and 24 total (40.7%) resections. After Propensity Score Matching (PSM), 35 patients remained in each group. The flowchart of the study is demonstrated in Figure 1.

**Figure 2:**
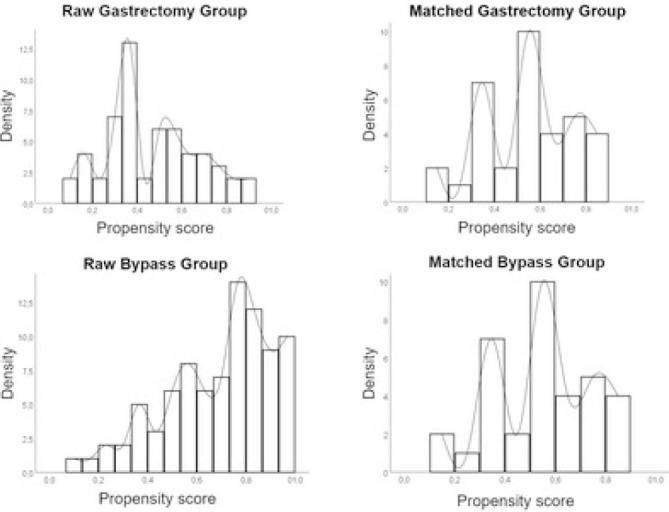
Histogram with the distribution of groups’ propensity scores before and after matching.

Table 1 summarizes the baseline data of the patients. Before PSM, patients in the bypass group had significantly more locally invasive tumors (p<0.01), more gastric outlet obstruction (p<0.01) and poorer clinical status as shown by the KPS (p=0.04), ASA (p=0.04), ECOG (p<0.01). The resected patients, on the other hand, had significantly more distant metastasis (p<0.01). After PSM, all baseline variables were similar except for GOOSS and cT (Figure 2).

**Table 1:** Clinical characteristics before and after Propensity Score Matching.

	Before PSM				p	After PSM				p
	Gastrectomy (n=59)		Bypass (n=91)			Gastrectomy (n=35)		Bypass (n=35)		
	n	%	n	%		n	%	n	%	
Sex										
Female	19	32.2	31	34.1	0.81	11	31.4	11	31.4	1
Male	40	67.8	60	65.9		24	68.6	24	68.6	
Age (years)										
<65	29	49.2	41	45.1	0.62	17	48.6	19	54.3	0.81
≥65	30	50.8	50	54.9		18	51.4	16	45.7	
BMI (kg/m²)										
<18.5	5	8.5	20	22	0.03	5	14.3	7	20	0.53
≥18.5	54	91.5	71	78		30	85.7	28	80	
cT										
0–3	15	25.4	0	0	<0.01	7	20	0	0	0.01
4	44	74.6	91	100		28	80	35	100	
cN										
N0	6	10.2	4	4.4	0.19	3	8.6	1	2.9	0.31
N+	53	89.8	87	95.6		32	91.4	34	97.1	
cM										
0	6	10.2	34	37.7	<0.01	6	17.1	5	14.3	0.74
1	53	89.8	57	62.6		29	82.9	30	85.7	
Metastatic sites										
1	47	79.7	41	45.1	<0.01	26	74.3	22	62.9	0.98
≥2	6	10.2	16	17.5		3	8.6	8	22.8	
CCI										
0	38	64.4	70	76.9	0.09	25	71.4	25	71.4	1
≥1	21	35.6	21	23.1		10	28.6	10	28.6	
ASA										
I–II	36	61	40	44	0.04	22	62.9	17	48.6	0.23
III–IV	23	39	51	56		13	37.1	18	51.4	
KPS										
<80	18	30.5	43	47.3	0.04	11	31.4	14	40	0.45
≥80	41	69.5	48	52.7		24	68.6	21	60	
ECOG										
0–1	43	72.9	45	49.5	<0.01	23	65.7	23	65.7	1
2–4	16	27.1	46	50.5		12	34.3	12	34.3	
GOOSS										
0–2	32	54.2	11	12.2	<0.01	15	42.9	5	14.3	0.01
3	27	45.8	79	87.8		20	57.1	30	85.7	
Albumin (g/dL)*										
<3.5	15	25.9	44	50.6	<0.01	11	31.4	12	34.3	0.8
≥3.5	43	74.1	43	49.4		24	68.6	23	65.7	
Hemoglobin (g/dL)										
<11	30	50.8	69	75.8	<0.01	21	60	21	60	1
≥11	29	49.2	22	24.2		14	40	14	40	
Preoperative chemotherapy										
No	49	83.1	73	80.2	0.66	27	77.1	26	74.3	0.78
Yes	10	16.9	18	19.8		8	22.9	9	25.7	

Obs.: Data not available for one patient from the gastrectomy group and four patients from the bypass group before PSM.

PSM: Propensity Score Matching; BMI: body mass index; cT: tumor; cN: lymphonode; cM: metastasis; CCI: Charlson Comorbidity Index; ASA: American Society of Anestesiology; KPS: Karnofsky Performance Status; ECOG: Eastern Cooperative Oncologic Group; GOOSS: gastric outlet obstruction scoring system.

Table 2 summarizes the postoperative outcomes after PSM. There were no significant differences in the incidence of complications up to 30 days after surgery, but the bypass group had a higher 90-day mortality. Both groups were able to start postoperative chemotherapy at a similar rate and received a similar number of palliative chemotherapy cycles.

**Table 2:** Outcomes of both groups after Propensity Score Matching.

	Gastrectomy (n=35;%)	Bypass (n=35; %)	p
POC (Clavien-Dindo)			
0–2	30 (85.7)	29 (82.9)	0.74
3–5	5 (14.3)	6 (17.1)	
Length of hospital stay (days)			
Median (IQR)	9 (6–14)	5 (4–8)	0.05
Mortality (day)			
30	2 (5.7)	6 (17.1)	0.25
90	3 (8.5)	14 (40)	<0.01
Postoperative chemotherapy			
Yes	26 (74.3)	21 (60)	0.20
No	9 (25.7)	14 (40)	
Nº of palliative chemotherapy cycles			
Median (IQR)	5 (2–7.25)	6 (2–9.5)	0.62
Overall survival (months)			
Median (IQR)	16.9 (6.9–23.8)	4.5 (1.6–9.5)	<0.01

POC: Postoperative complications; IQR: Interquartile range.

**Figure 3:**
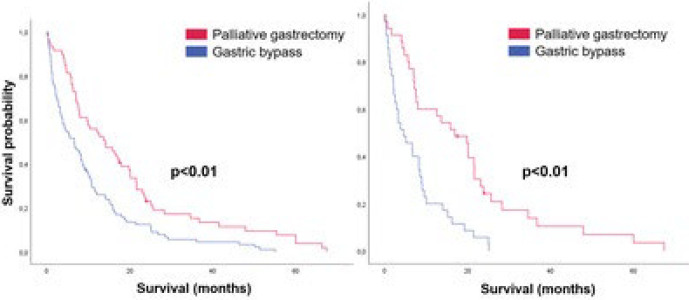
Overall survival curves before and after Propensity Score Matching.

Figure 3 illustrates the Kaplan-Meier curves before and after PSM. Before matching, OS for the gastrectomy group was 14.1 months and 6.6 months for the bypass group (p<0.01). After PSM, the difference remained, with resected patients having an OS of 16.9 months and bypassed patients having an OS of 4.5 months (p<0.01).

Table 3 illustrates variables associated with survival. After univariate analysis, cM, GOOSS, preoperative albumin, surgical technique, and postoperative chemotherapy were significantly associated. However, after multivariate analysis, only preoperative albumin (HR=2.02; 95%CI 1.17–3.48; p=0.01), postoperative chemotherapy (HR=5.97; 95%CI 3.03–11.7; p<0.01) and surgical technique (HR=3,28; 95%CI 1.8–5.95; p<0.01) were associated with survival.

**Table 3:** Univariate and multivariate analysis of variables associated with survival.

Variables	Univariate			Multivariate		
	HR	95%CI	p	HR	95%CI	p
Sex (male *vs.* female)	0.77	0.45–1.31	0.35	-	-	-
Age (<65 *vs.* ≥65 years)	0.73	0.44–1.19	0.21	-	-	-
cM (M0 *vs.* M+)	2.37	1.16–4.84	0.01	1.89	0.90–3.96	0.09
cN (N0 *vs.* N+)	2.93	0.90–9.49	0.07	1.54	0.44–5.30	0.49
cT (T1-3 *vs*. T4)	0.94	0.40–1.99	0.8	-	-	-
CCI (0 *vs.* ≥1)	0.85	0.49–1.46	0.56	-	-	-
ECOG (0–1 *vs.* 2–4)	1.57	0.94–2.62	0.08	1.10	0.62–1.94	0.73
GOOSS (0–2 *vs.* 3)	1.75	1.02–3.00	0.04	1.21	0.65–2.24	0.54
Albumin (≥3.5 *vs.* <3.5 g/dL)	1.80	1.08–3.02	0.02	2.02	1.17–3.48	0.01
BMI (≥18.5 *vs.* <18.5 kg/m²)	0.86	0.46–1.62	0.65	-	-	-
Adjuvant chemotherapy (yes *vs.* no)	4.43	2.49–7.88	<0.01	5.97	3.03–11.7	<0.01
Surgery (Gastrectomy *vs.* Bypass)	2.83	1.67–4.80	<0.01	3.28	1.80–5.95	<0.01

HR: hazard ratio; CI: confidence interval; cM: metastasis; cN: lymphonode; cT: tumor; CCI: Charlson Comorbidity Index; ECOG: Eastern Cooperative Oncologic Group; GOOSS: gastric outlet obstruction scoring system; BMI: body mass index.

## DISCUSSION

Patients with clinical stage IV GC who underwent palliative gastrectomy were compared with patients undergoing bypass without resection of the primary tumor. After performing PSM, it was found that patients in the gastrectomy group had better survival without increased operative mortality.

Theoretically, tumor resection, even in the setting of an incurable disease, is thought to somewhat improve disease control by the reduction of tumor burden. This reduces not only cytokines produced by the primary tumor that led to systemic symptoms, but the number of tumor stem cells, therefore enhancing chemotherapy sensitivity^18,19^. Besides, it grants rapid resolution of symptoms related to local invasion. This has been proven helpful for tumors such as ovarian and colorectal cancer^22^. In that sense, palliative gastrectomy as a treatment option for incurable GC has long been studied. GC is a biologically aggressive tumor, and even with standard palliative chemotherapy, survival for clinical stage IV patients is around ten months^18^.

For asymptomatic patients, reports have conflicting results as to whether tumor resection is a safe and efficient treatment^15,16,18,23,24^. The REGATTA trial, a phase III clinical trial, compared the survival gain of cytoreductive gastrectomy followed by chemotherapy to chemotherapy alone in asymptomatic patients with advanced GC and the resection was not associated with longer survival^8,22^. Within this context, nowadays resection in asymptomatic patients is only indicated in the context of a conversion surgery when R0 resection is achievable.

For patients presenting with complications related to the primary tumor such as bleeding, perforation, or obstruction, chemotherapy alone does not provide adequate control of the symptoms. In those situations, surgical treatment is required^9,10^. The question here relies on which invasive approach is best, both in terms of surgical morbidity and overall survival. This doubt is particularly difficult when gastric outlet obstruction is present.

Most concerns about the indication of palliative gastrectomy are due to its high mortality, with reports from up to the mid-1990s sometimes reaching 20%^14^. There is no doubt that this is a fragile group of patients, often with associated malnutrition and anemia. The description of the initial clinical characteristics of the patients in the present study corroborates this statement. However, progress in surgical technique and perioperative care has diminished this rate to less than 5% in recent studies^6,13,14,26^. In our study, palliative gastrectomy had a 30-day mortality rate of 5.7%. This value is higher than the rate of 3.3% reported by our service for curative intent gastrectomy with D2 lymphadenectomy and inferior to the 17.8% rate of patients who underwent D1 lymphadenectomy due to unfavorable clinical conditions^27,28^. Therefore, we considered the mortality rate of the present study acceptable considering the severity of the included patients.

Patients undergoing bypass had a higher frequency of locally advanced T4b tumors, which was the major factor that prevented their resection. Although we performed the matching with resected patients with similar clinical characteristics, it is undeniable that this was a group of patients with more advanced tumors. However, we were surprised by the high mortality within 90 days of this group since it is an easy-to-perform, low-complexity procedure. This may be because the bypass group had a lower local disease control, and therefore a higher probability of recurrent onset of systemic and local symptoms such as vomiting and obstruction, impairing adequate nutrition and bringing the risk for clinical complications such as aspiration pneumonia.

Our finding regarding the longer survival for resected patients is in agreement with other reports^6,15,24,32,33^. However, some other studies have shown different results. Okumura et al. found that gastrectomy did not enhance survival when compared to bypass in the setting of gastric obstruction, but this retrospective study only had a sample size of 43, not matched for selection biases^23^. Chen et al. showed that there was no difference in survival even when using a larger sample (n=199) and after performing PSM^4^. The diversity of results may be linked to multiple factors that vary amongst reports, such as length of resection, baseline patients’ characteristics and if chemotherapy was possible after surgery. Reports with a larger sample that could allow for subgroup analysis would help in evaluating the subset of patients that benefit the most from resection.

Some limitations of our study should be discussed. First, it is a single center retrospective study. Second, due to the sample size, subgroup analysis of distal and proximal lesions was not possible. Finally, there was no standardization on the chemotherapy regimen used. Despite these shortcomings, the study comprises a considerable cohort of well-characterized patients, and analysis was performed after propensity score matching for balancing baseline characteristics.

In summary, palliative gastrectomy, when feasible, seems to improve survival without increasing morbidity in symptomatic clinical stage IV GC patients. However, the criteria for selecting patients that benefit the most from the resection are still to be identified.

## CONCLUSIONS

Clinical stage IV GC often presents with symptoms that require surgical intervention. Palliative gastrectomy was associated with longer survival and lower perioperative morbidity when compared to bypass after PSM. This may be due to better local disease control, granting lower rates of clinical complications and better chemotherapy effectiveness.
